# Facilitators and barriers to detection and treatment of depression, anxiety and experiences of domestic violence in pregnant women

**DOI:** 10.1038/s41598-023-36150-z

**Published:** 2023-08-01

**Authors:** Zulfa Abrahams, Sonet Boisits, Marguerite Schneider, Simone Honikman, Crick Lund

**Affiliations:** 1 Department of Psychiatry and Mental Health, Alan J Flisher Centre for Public Mental Health, University of Cape Town, Building B, 46 Sawkins Road, Rondebosch, Cape Town, 7700 South Africa; 2 Perinatal Mental Health Project, Department of Psychiatry and Mental Health, Alan J Flisher Centre for Public Mental Health, University of Cape Town, Cape Town, South Africa; 3 Health Service and Population Research Department, King’s Global Health Institute, Centre for Global Mental Health, Institute of Psychiatry, Psychology and Neuroscience, King’s College London, London, UK

**Keywords:** Health services, Public health, Health care, Risk factors

## Abstract

In South Africa, symptoms of common mental disorders (CMDs) such as depression and anxiety are highly prevalent during the perinatal period and linked to experiences of domestic violence. However, limited routine detection and treatment is available to pregnant women with these problems, even though evidence suggests that screening and treating CMDs during pregnancy improves the health and economic outcomes of mothers and their children, and has been suggested as a key approach to improving the health of perinatal women and children. We investigated facilitators and barriers of service-providers and service-users in detecting and treating pregnant women with symptoms of CMDs and experiences of domestic violence. This study was conducted in four midwife obstetric units (MOUs) in Cape Town, South Africa, and in the non-profit organisations providing community-based support in the communities surrounding the MOUs. Service-provider perspectives were informed by qualitative interviews with 37 healthcare workers providing care to pregnant women. Qualitative interviews with 38 pregnant women attending the same MOUs for their first antenatal care visit provided service-user perspectives. Facilitators identified included the availability of a mental health screening questionnaire and the perceived importance of detection and treatment by both service-providers and -users. Barriers contributing to the low detection rates included service-users concerns about the lack of confidentiality and feelings of shame related to experiences of domestic violence as well as service providers discomfort in dealing with mental health issues, their limited time available and heavy patient load. In addition, service-providers highlighted the lack of standardised referral pathways and the poor uptake of referrals by women with symptoms of depression and anxiety, or experiences of domestic violence. While the system-level barriers need to be addressed at a policy level, the patient- and provider-level barriers identified indicate the need to strengthen health systems by training antenatal care nurses to detect symptoms of CMDs and experiences of domestic violence in pregnant women, developing standardised referral pathways and training lay healthcare workers to provide treatment for mild symptoms of depression and anxiety.

## Introduction

Common mental disorders (CMDs), such as depression and anxiety, are highly prevalent during the perinatal period, with low- and middle-income countries (LMIC) carrying the greatest burden. It is estimated that in LMIC, 18% of perinatal women experience depression^1^ and 34% experience anxiety^2^. In South Africa, the prevalence of depressive symptoms during pregnancy ranges between 27 and 39%^3–6^, while symptoms of anxiety range between 15 and 23%^3,7^. In addition, South African studies indicate a high prevalence of domestic violence and food insecurity during the perinatal period which has been shown to increase the risk of CMDs^6,10^. Studies report an approximate 15% prevalence of domestic violence^8,9^ and a 40% prevalence of food insecurity^6^ amongst pregnant women living in low resource settings in Cape Town. Antenatal CMDs is associated with adverse infant and child outcomes, such as preterm birth, low birth weight, infant stunting and underweight children^11–13^. In LMIC, evidence suggests that screening and treating CMDs during pregnancy improves the health and economic outcomes of mothers and their children^14,15^, and has been suggested as a key approach to improving the health of perinatal women and children in South Africa^16^.

As a result of a growing awareness of the burden of disease and economic costs associated with mental disorders, South Africa adopted the Mental Health Policy Framework and Strategic Plan 2013–2020^17^ which aligns with the WHO Mental Health Action plan^18^. However, having a new policy framework supporting the integration of mental health into primary healthcare^19^ has not led to the desired changes in service delivery, and the detection of symptoms of CMDs continue to be excluded from routine care provided at public healthcare facilities in South Africa^20^.

Little is known about the facilitators and barriers to detection, referral and treatment of pregnant women with depression, anxiety and experiences of domestic violence in public sector healthcare facilities in South Africa. The Health System Strengthening in sub-Saharan Africa (ASSET) study^21^ has undertaken to work with the Western Cape Department of Health (DoH) to develop and evaluate the impact of a screening, referral and counselling intervention for pregnant women with CMDs and experiences of domestic violence. The ASSET study consisted of three phases—a pre-implementation, development and implementation phase. Using data collected during the pre-implementation phase, this study investigated the facilitators and barriers to detecting and treating pregnant women with symptoms of CMDs and experiences of domestic violence attending public sector healthcare facilities in Cape Town.

## Methods

### Setting

In South African primary healthcare (PHC) facilities, antenatal care is provided by nursing staff as well as lay healthcare workers, like HIV and breastfeeding counsellors. The DoH also funds non-profit organisations (NPOs), mandated to provide facility- and community-based support to patients attending PHC facilities. NPOs employ teams of lay community health workers (CHWs) who are managed by professional nurses, called outreach team leaders (OTL).

This qualitative study was conducted in midwife obstetric units (MOUs) and in the NPOs providing community-based support to them. All MOUs are situated on the ‘Cape Flats’^22^—a large, flat area covered by sandy soil on the outskirts of the city of Cape Town, and offer free antenatal, birthing and postnatal services to pregnant women. The area consists of Black and Coloured townships where these communities were forcibly moved to because of Apartheid laws in South Africa. Coloured people are a distinct cultural group whose ancestry includes Khoi and San peoples as well as slaves imported from Batavia, Madagascar, Indonesia, Malaysia, India, Angola and Mozambique^23^. Many years after the Apartheid laws were abolished, South Africans continue to see themselves in the racial categories assigned to them during Apartheid, with many continuing to experience unequal distribution of income and opportunities^24^, resulting in high levels of unemployment, poverty^25^, substance abuse^26^, gang violence^27^ and domestic violence^8^ being prevalent in their communities.

### Data collection

This study used both quantitative and qualitative data collection methods. Quantitative data collection was used to collect ‘fact-based’ information from the pregnant women, while the qualitative data collection was used to assess the thoughts and feelings of the healthcare workers and the pregnant women. Quantitative data was collected from pregnant women attending the MOU for their first antenatal clinic appointment and consisted of research assistants administering several validated and bespoke questionnaires to screen them for symptoms of CMDs, psychological distress and domestic violence.

Qualitative data collection, using key informant interviews, were used to collect information from (1) healthcare workers regarding their role and attitude to detecting, referring and treating pregnant women with symptoms of depression or anxiety, and experiences of domestic violence; and (2) to pregnant women regarding their understanding of depression, anxiety and experiences of domestic violence, and their perceived reasons for experiencing psychological distress.

Healthcare workers providing care to pregnant women, working in four MOUs or the supporting NPOs, were purposively selected and recruited between August and December 2019. Purposive selection was used to ensure that the following cadres of healthcare workers involved in the care of pregnant women, were invited to participate in semi-structured interviews: operational managers, antenatal care (ANC) nurses, breastfeeding counsellors, HIV counsellors, health promotion officers, mental health nurses, social workers, CHWs and OTLs. All except one health promotion officer agreed to participate in the study.

During November 2019 and June 2020, pregnant women attending MOUs for their first antenatal appointment were invited to participate in the study. Healthcare workers (who were not part of the study), handed all pregnant women attending the MOU for their first antenatal clinic appointment a copy of the study information sheet and briefly explained what the study was about. After 30 min or more, a research assistant approached the women and explained the study in more detail, before asking them to provide informed consent. For women who were younger than 16 years, parental consent in addition to the minor’s assent was obtained. Women were offered a R100 grocery voucher, to compensate for the time taken to participate in the study. Those who consented were screened for symptoms of CMDs using the Edinburgh Postnatal Depression Scale (EPDS)^28^ and a psychological distress questionnaire^29^. In addition, participants were screened for experiences of domestic violence, using a bespoke questionnaire with a 12-month recall period and asked about experiences of verbal or emotional abuse (e.g., shouting or swearing, name calling, insulting, threats of intimidation, ignoring or excluding, isolating, humiliating, blaming), physical abuse (e.g., beating, pushing, kicking, biting, slapping) and sexual abuse (e.g., forced sexual intercourse or intercourse without your consent). The EPDS, using a 7-day recall period, has been validated in South Africa with a cut-off ≥ 13 indicating a probable CMD^28,30,31^. The psychological distress questionnaire comprised three mood questions^29^, derived from the Whooley scale^32^, the Generalised Anxiety Scale (GAD-2)^33^, and the EPDS^28^, uses a two-week recall period, and was validated (in South Africa) against the EPDS. It was found to correctly classify 91% of women using a cut-point of ≥ 2 (sensitivity = 85.7; specificity = 92.9)^29^. Pregnant woman who scored ≥ 13 on the EPDS, ≥ 2 on the psychological distress questionnaire or ≥ 2 on the domestic violence questionnaire were invited to participate in a semi-structured interview. Recruitment continued at all facilities until saturation was reached.

English language, semi-structured interview guides were developed, and translated to Afrikaans and IsiXhosa by bilingual experts. The interviews were administered by trained researchers in English (with a PhD), Afrikaans (with a Bachelor’s degree) and IsiXhosa (without a tertiary qualification, but having extensive experience in qualitative data collection), took between 30 and 60 min to complete, and were audio-recorded. All interviews were conducted in a private space, with no others present, to ensure confidentiality. Healthcare workers were asked about their role and attitude to detecting, referring and treating pregnant women with symptoms of depression or anxiety, and experiences of domestic violence (Supplementary File 1). Pregnant women were asked about their understanding of depression, anxiety and experiences of domestic violence, and their perceived reasons for experiencing psychological distress (Supplementary File 2). All participants were asked about their thoughts on the acceptability and feasibility of a routine screening and counselling service for pregnant women.

### Data analysis

The semi-structured interviews that were conducted in English were transcribed verbatim, while interviews conducted in Afrikaans or IsiXhosa were translated into English and transcribed by professional translators/transcribers. A type of thematic analysis approach, known as framework analysis^34^, were used to analyse the transcripts. The framework approach comprises seven interconnected stages that provide clear guidance on data analysis, allowing for the application of a framework of key themes guiding an enquiry, along with the emergence of new themes from the data. These stages include transcription, familiarisation, coding, developing a working analytical framework, applying the framework, charting and interpretation. This approach is commonly used for the analysis of semi-structured interviews in health systems and health policy research, and consists of categorising commonalities using codes and themes, which is then displayed as a matrix, allowing the researcher to analyse the data in a systematic way. The development of initial codes was guided by the semi-structured interview topics. Further themes not captured by the initial coding were identified through extensive reading of the transcripts and coding passages interpreted as important. Transcripts and data were managed using NVivo 12 Pro qualitative data analysis software (QSR International Pty Ltd)^35^.

Ten percent of the healthcare workers transcripts were randomly selected for analysis by three researchers, and the inter-rater reliability assessed (agreement = 78.4%; Gwet’s agreement coefficient (AC)^36,37^ = 0.7329). Coding disagreements were resolved by discussing the reasons for assigning a specific code and agreeing on the most appropriate code to use. Thereafter, each of the three researchers analysed a third of the remaining transcripts.

Similarly, 10% of the pregnant women’s transcripts were randomly selected for analysis by two researchers, and the inter-rater reliability assessed (agreement = 87.3%; Gwet’s AC = 0.8547). Coding disagreements were discussed and resolved. Thereafter, each researcher analysed half the remaining transcripts.

### Implementation science

Using data collected during the pre-implementation phase of the ASSET study^38^, implementation science determinant frameworks were used to identify the facilitators and barriers to detecting and treating pregnant women with symptoms of CMDs and experiences of domestic violence. The Theoretical Domains Framework (TDF)^39^ and the Context and Implementation of Complex Interventions Framework (CICI)^40^ were used to identify determinants that could potentially influence the implementation of the ASSET intervention.

### Ethical approvals

Ethical approval for the study was obtained from the Human Research Ethics Committee at the University of Cape Town (Ref No: 139/2018) and the Psychiatry, Nursing and Midwifery Research Ethics Subcommittee at King’s College London (Ref No: 17/18-7807). In addition, the Western Cape Department of Health approved the use of the research sites (Ref No: WC_201807_008). All relevant guidelines, procedures and regulations were followed. Those who participated in the study provided written, informed consent after the procedure had been verbally explained in their home language (Afrikaans, English or IsiXhosa). All participants were informed that they were free to withdraw from the study at any time without consequences.

## Results

Semi-structured interviews were conducted with 37 healthcare workers who provided care to pregnant women or managed staff providing the care (Table 1). The majority of the healthcare workers were female (91.9%), older than 35 years (75.7%), employed by an NPO (56.8%) and worked at an MOU (67.5%).

**Table 1:** Demographic characteristics of healthcare workers (n = 37).

Characteristics	Healthcare workers n (%)
Gender	
Female	34 (91.9)
Male	3 (8.1)
Age	
Under 25 years	2 (5.4)
26–35 years	7 (19.0)
36–50 years	15 (40.5)
Over 50 years	13 (35.1)
Race	
White	3 (8.1)
Coloured*	28 (75.7)
Black	6 (16.2)
Healthcare Worker Role	
Manager	13 (35.1)
Professional healthcare worker	10 (27.0)
Lay healthcare worker	14 (37.9)
Employer	
Department of health	16 (43.2)
Non-profit organisation (NPO)	21 (56.8)
Employment environment	
Facility-based	25 (67.5)
Community-based	12 (32.5)

*Individuals of mixed ancestry^20^.

One hundred and fifty-six pregnant women were screened for symptoms of CMDs and experiences of domestic violence. Thirty-eight women (24%) screened positive and were invited to participate in a semi-structured interview (Table 2). The majority of women interviewed were 26 years or older (55.2%) and were unemployed (44.7%). Almost all the women interviewed scored ≥ 2 on the psychological distress questionnaire (94.7%), while 29% (n = 11) scored ≥ 2 on the domestic violence questionnaire.

**Table 2:** Demographic and clinical characteristics of pregnant women (n = 38).

Characteristics	Pregnant women n (%)
Age	
15–18 Years	6 (15.8)
19–25 years	11 (29.0)
26–35 years	17 (44.7)
36–40 years	4 (10.5)
Race	
Coloured*	26 (68.4)
Black	12 (31.6)
Employment status	
Employed	12 (31.6)
Unemployed	17 (44.7)
Student	9 (23.7)
Gestation	
First trimester	15 (39.5)
Second trimester	19 (50.0)
Third trimester	4 (11.5)
Previous pregnancies	
None	10 (26.3)
1–3 pregnancies	21 (55.3)
> 3 pregnancies	5 (18.4)
Scored ≥ 13 on EPDS**	24 (63.2)
Scored ≥ 2 on the mental health screening questionnaire	36 (94.7)
Scored ≥ 2 on domestic violence screening questionnaire	11 (29.0)

*Individuals of mixed ancestry^20^.

**Edinburgh Postnatal Depression Scale.

The facilitators and barriers identified by healthcare workers and pregnant women have been organised into three categories: system-level, provider-level and patient-level (Table 3). The facilitators and barriers will be presented under two main headings representative of the stages along the care pathway: (1) detection, and (2) referral and treatment.

**Table 3:** System-, provider- and patient-level facilitators and barriers to detection, referral and treatment for common mental disorders (CMDs) and experiences of domestic violence.

	Facilitators	Barriers
Detection of CMDs and experiences of domestic violence		
Availability of a mental health screening questionnaire	System-level	
Aspects of the mental health screening questionnaire		System-level
Lack of a domestic violence screening questionnaire		System-level
Perceived importance of detection	Provider-level Patient-level	
Perceived lack of confidentiality		Patient-level
Heavy workload		Provider-level
Discomfort with mental health issues		Provider-level
Referral and treatment of CMDs and experiences of domestic violence		
Lack of availability of referral pathways		System-level
Poor awareness of referral pathways		Provider-level Patient-level
Perceived importance of counselling	Provider-level Patient-level	
Cultural beliefs and stigma		Patient-level

### Detection

#### Tools for detection

Varying rates of antenatal mental health and domestic violence screening were reported across the four facilities. The Perinatal Mental Health Project (PMHP)^41^ supported one of the facilities by providing an on-site screening and counselling service to all pregnant women attending the MOU for their first antenatal visit. As part of the service, a health promotion officer screened all pregnant women at their first antenatal visit for symptoms of CMDs and the risks thereof. Women who screened positive were referred to the on-site counsellor. The PMHP reported detecting many women with symptoms of depression and anxiety—*“I guess six out of ten*” (Health Promotion Officer), while the other facilities detected fewer women—*“I see two or three on a weekly basis”* (Health Promotion Officer*)*, and *“It’s very rare…”* (ANC Nurse). During data collection, an updated version of the Maternity Case Record (MCR)^42^—the national stationery used to record all aspects of the pregnancy—was released. The updated MCR contained a mental health screening tool^29^ (the same questionnaire used in this study to detect psychological distress), to be administered by ANC nurses at the patients’ first clinic visit.

Detecting women with experiences of domestic violence was less common in all facilities. Healthcare workers reported that they did not specifically enquire about domestic violence and only detected it when a physical examination revealed signs of physical abuse, or when the woman voluntarily disclosed the information. One ANC nurse reported detecting women who were abused “*twice or thrice a month*”, while an MOU manager reported that detecting women who were abused was “*not often, but we do*”. A health promotion officer admitted that if she specifically enquired about domestic violence, she would detect many women as “*domestic violence is so high here in this area*”.

#### Perceived importance of detection

All except for one of the facility-based healthcare workers interviewed felt that it was important to screen pregnant women for psychological distress and experiences of domestic violence. One mental health nurse made the link between psychological distress that was left untreated and the consequences to the mother and child by saying: *“I think it's very important that these things needs to be picked up because it can only get worse when the baby is born”.* Many healthcare workers felt that screening should occur at all clinic visits. One ANC nurse mentioned that CMDs could occur at any point during the perinatal period, not just when women attended the MOU for the first time as psychological distress *“can start any time during pregnancy”.*

#### Perceived lack of confidentiality

While the majority of pregnant women reported that they would be happy to disclose their feelings and experiences of domestic violence, a few women expressed their concerns regarding confidentiality. One pregnant woman was especially concerned about whether such sensitive information would remain confidential by saying: *“… especially in a community where everyone knows everyone, I do not trust them* [nurses]*”*. Similarly, some healthcare workers expressed doubts about whether the pregnant women would disclose their feelings by saying: *“… remember people put up a nice face hey, they don’t want people to know how they feeling”* (Breastfeeding Counsellor).

In the context of the four facilities, many women were financially dependent on their partners, and were concerned about disclosing their experiences of domestic violence. A breastfeeding counsellor explained why women who were abused rarely spoke about their experiences by saying: *“Some of them are ashamed that they're actually having to tell you this”,* while a pregnant woman referred to the possible consequences of disclosing her abuse by saying *“… so we have to think twice before you do something that you are going to regret”*.

#### Heavy workload

All ANC nurses were concerned about the amount of time it took to assess patients’ mental health as their heavy workload and large patient numbers allowed them limited time with each patient. One ANC nurse highlighted her heavy workload in relation to her role as a midwife by saying: “*I can't* [screen all patients] *because now it's gonna take me a long time… then you worry, I need to see the next patient.…. so I won't be giving much time to her. I will only just be very…just so, just on the top”.* In addition, ANC nurses reported feeling concerned that they would not be able to provide a comprehensive consultation if they had the added responsibility of asking women about their mental health. One ANC nurse chose to wait for patients to mention that they were feeling distressed, instead of administering the screening tool to all patients—“*if she’s not gonna ask you or tell you about her problem then you oversee that—you overlook that, because she looks happy, she’s not looking stressful.*” Many of the ANC nurses were also concerned about the amount of time it took to contain a patient who felt distressed as a result of administering the screening tool. One ANC nurse reflected on her experiences by saying: *“sometimes they will start opening up and then it's a problem. You open a can of worms. Cause those questions are thought-provoking.…if they're gonna start crying…and then for me it sounds rude to just okay, I'm gonna send you to ….”*

#### Discomfort with mental health issues

Many ANC nurses felt uncomfortable discussing mental health issues with patients. They thought of themselves as clinicians who were primarily responsible for the physical well-being of the pregnant woman and her foetus, and that they were not trained to adequately address mental health issues. One ANC nurse described the discomfort she experienced when discussing issues related to mental health by saying: “*I can talk and everything but there's a point where right, I'm not trained for this … I don't want to take in so much because it also takes so much out from you*”. Other healthcare workers were concerned that they were not trained to manage women who screened positive—“*if you probe and you ask those three questions* [the screening tool]*, what do you do if the answer is yes?”* (ANC Nurse).

### Referral and treatment

#### Lack of availability of referral pathways

Referral pathways varied across the four facilities and depended on the issue requiring referral. Patients with depression or anxiety were referred to the mental health nurse, a social worker, a registered psychologist (if available) or to the district hospital, while those who felt suicidal were referred to the mental health nurse, a medical officer, the trauma unit or to a registered psychologist. Women who reported experiencing domestic violence were most often referred to the social worker, if one was available. However, specialists such as social workers, mental health nurses and registered psychologists carried a heavy workload and could often only see women who were referred a few weeks later. While some healthcare workers were aware of the referral pathway in their facility, many were not. One lay healthcare worker voiced her uncertainty by saying: “*I do not even sometimes know where to refer the mothers to”* (HIV Counsellor)*.* The majority of pregnant women were also unaware of how to access mental health care, or whether it was available at the healthcare facilities.

#### Perceived importance of counselling

Both healthcare workers and pregnant women thought that providing a counselling service was important. A healthcare worker expressed her thoughts on the importance of counselling by saying: *“… many women who are untreated, or undiagnosed or not caught in the system do end up later on in life here at psychiatry or even with worse symptoms and now it is not only affected themselves but now also the baby after its being born”* (Mental Health Nurse)*.* Similarly, a pregnant woman agreed that counselling was important by saying: *“I think it would be good, yes, because talking about it makes you feel much better and for me talking to a stranger is much better than talking to people that knows you”.*

#### Cultural beliefs and stigma

While the importance of providing a counselling service was highlighted, healthcare workers also pointed out that many women who were referred for mental health counselling, declined the offer. One ANC nurse explained that mental health issues were stigmatised in the community and that patients were concerned that they would be branded as “…*mad if you go for counselling”.* A lay healthcare worker explained that the community was very small, and that patients were concerned that *“… they* [neighbours] *look at others and they point fingers”* (CHW)*.*

Healthcare workers blamed the normalisation of psychological and physical abuse in the community for the poor uptake of referrals by women who were abused, whereas pregnant women attributed their financial dependence on their partner or husband. Healthcare workers thought that women who were abused believed that the abuse was “… *how he is showing his love*” (Breastfeeding Counsellor), or that he *“did not mean to hurt them*” (ANC Nurse), or that the women “*deserve it*” (Breastfeeding Counsellor). A pregnant woman verified the normalisation of domestic violence in her community by saying: *“… it* [the abuse] *is not a big thing for them* [family and friends], *because it happens a lot*”. Many pregnant women who were abused felt that a referral for mental health counselling would not help them as it would not be able to change their dependence on their abusive partners. One pregnant woman explained that she had accepted her abusive relationship by saying: “*Everyone mos* [anyway] *has their own problems …*”, while another pregnant woman explained that counselling could not change her situation as “*I am not working. He is the breadwinner*”.

## Discussion

This paper utilizes qualitative semi-structured interviews with healthcare workers and pregnant women, to identify the facilitators and barriers to mental health care for pregnant women attending MOUs in Cape Town. While healthcare workers and pregnant women acknowledged the importance of detecting women with symptoms of depression and anxiety, or experiences of domestic violence, detection rates were low. Barriers contributing to the low detection rates included patients’ concerns about the lack of confidentiality and feelings of shame related to experiences of domestic violence; and healthcare workers discomfort in dealing with mental health issues, their limited time available and heavy patient load. Interviews with healthcare workers highlighted the lack of standardised referral pathways and the poor uptake of referrals by women with symptoms of depression and anxiety, or experiences of domestic violence.

Several system-, provider- and patient-level facilitators and barriers were identified. The availability of a mental health screening tool, included in the updated MCR^42^ was identified as a *system-level facilitator*, as it enabled nurses to screen pregnant women for CMDs as part of their routine consultations. However, several *system-level barriers* linked to the MCR were also identified: the screening tool contains a caveat, indicating that the screening should only be conducted if “resources are available for referral, e.g. mental health nurse, social worker, NPO, medical officer, counsellor, psychiatrists or other services”; the MCR only allows for the screening tool to be completed at the first consultation; and no questions are available to detect experiences of domestic violence^42^. As depression and anxiety may develop^8^ and remit^43^ at any point during the perinatal period, only administering the screening tool at the first clinic visit, would result in missed opportunities for care for women who develop symptoms of depression or anxiety later in their pregnancy, or after birth. While the MCR provides a list of counselling topics, including one for domestic violence, the lack of a screening question for detecting domestic violence could be responsible for the poor detection rates found in communities where domestic violence is prevalent^8,44^, as studies suggest that routine screening for domestic violence in primary healthcare settings improves detection^45,46^. The final *system-level barrier* contributing to the low detection and referral rate, was the lack of standardised referral pathways. We found that both healthcare workers and pregnant women were unclear about how and where to access care. As the mental health screening tool was only to be conducted when resources were available, healthcare workers who were unaware of resources available in the facility, were free to opt out of providing the screening service to perinatal women.

*Provider-level facilitators* included the perceived importance of detecting pregnant women with CMDs and experiences of domestic violence, and the treatment of women who screened positive. In this study, healthcare workers acknowledged the importance of providing a screening, referral and counselling service. However, several *provider-level barriers* were identified. ANC nurses who were tasked with conducting the screening felt that they were not the right cadre of staff to provide the service, citing their large patient load, the additional time needed to screen patients, their lack of awareness of the referral pathway, and their discomfort with mental health issues as the primary reasons for their reluctance. Studies investigating the challenges to providing quality healthcare in South Africa have highlighted the shortage of healthcare workers, especially at the nursing level in urban areas^47^. As it is unconstitutional to deny anyone access to basic healthcare in public healthcare facilities in South Africa^48^, these facilities are often overcrowded with inadequate staff and resources available to provide quality care to all^47^. However, this situation is not unique to South Africa. In Zimbabwe, provider-level barriers to screening perinatal women attending PHC facilities for depression included nurses’ lack of time and inadequate training, and lack of privacy making patients less likely to disclose psychological distress^49^. While the lack of privacy was not identified as a barrier in the facilities included in this study, it is a challenge in other PHC facilities in South Africa^50^. Similar findings were reported in a review of studies exploring healthcare workers perceived barriers to screening, referral and management of mental health issues in perinatal women^51^, and in reviews of barriers to IPV screening as perceived by healthcare providers^52–54^. Studies highlighted time constraints, lack of knowledge and training, and insufficient awareness of referral pathways as key provider-level barriers to screening.

*Patient-level facilitators* included the perceived importance of screening and counselling, while the* patient-level barriers* included concerns regarding confidentiality when disclosing sensitive information. High levels of mental health stigma have been reported in South Africa. In a qualitative study exploring the experiences of mental health stigma among healthcare providers and users in the North West province of South Africa^55^, the authors reported that users were exposed to stigmatising attitudes of family members, neighbours, friends, church members and the general community. Similarly, a study in Massachusetts USA reported that perinatal women were reluctant to disclose their mental health issues due to stigma and the fear of being judged as an unfit parent, resulting in missed appointments and poor uptake of services^52^.

Our study has strengths and limitations. We have both the perceptions of service providers and service users from the same clinics. However, our service users were interviewed when attending the clinic for their first antenatal appointment, making it difficult to link their perceptions of care with the specific healthcare providers we interviewed. Our study may be limited by social desirability bias, especially regarding the pregnant women’s responses to questions on domestic violence, and service providers’ responses to their role in providing care. In addition, we did not ask service providers how they managed the feelings of stigma among their patients.

Several strategies can be used to mitigate the challenges we have identified and strengthen the health system. Importantly, strategies for addressing barriers in pregnant women should be addressed differently to barriers in healthcare workers. ANC nurses require training on administering the screening tool to decrease their feelings of discomfort with mental health issues. As nurses’ time with patients is limited, the mental health screening questionnaire could be administered in a patient centred manner^56^. ANC nurses could be trained to routinely enquire about their patients’ feelings and anxieties while examining them, instead of completing the screening tool as a ticking exercise while completing the required documentation linked to the consultation. This method of assessment would address the ANC nurses concern regarding the time needed to screen patients, as well communicate a genuine interest in patients’ emotional well-being. Standardised referral pathways and processes, specific to each facility, need to be developed and disseminated widely to ensure that both healthcare workers and patients are aware of the services available and how to access them. To lessen the burden of specialised mental healthcare providers such as social workers and mental health nurses, a cadre of lay healthcare workers could be identified and trained to provide basic evidence-based problem-solving counselling^57^ to women with mild symptoms of depression or anxiety. The limitations of the MCR will need to be addressed at a policy level to encourage healthcare providers to detect symptoms of depression, anxiety and experiences of domestic violence in all pregnant women, at all clinic visits. Intervention studies aimed at strengthening the healthcare system with regards to detection, referral and treatment at MOUs in South Africa are needed.

## Conclusions

Facilitators identified by healthcare workers and pregnant women included the importance of detecting women with symptoms of CMDs and experiences of domestic violence and the availability of a mental health screening tool, yet detection rates at the MOUs were low. Several barriers were identified—at the system-level, these related to aspects of the screening tool, and the lack of a screening questionnaire to identify experiences of domestic violence. Provider-level barriers included healthcare workers discomfort in dealing with mental health issues, their limited time available, heavy patient load and lack of awareness of available referral pathways. Patient-level barriers included patients’ concerns about the lack of confidentiality, and feelings of shame related to experiences of domestic violence. While the system-level barriers need to be addressed at a policy level, the patient- and provider-level barriers will be used by the ASSET study to inform the development of a health systems strengthening intervention, to be piloted in MOUs and basic antenatal care clinics in Cape Town.

## Supplementary Information

Supplementary Information 1.

Supplementary Information 2.

## Data Availability

The datasets used and/or analysed during the current study are available from the corresponding author on reasonable request.

## Abbreviations

ANC: Antenatal care
ASSET: Health system strengthening in sub-Saharan Africa
CHW: Community health worker
CMD: Common mental disorders
DoH: Department of Health
EPDS: Edinburgh postnatal depression scale
LMIC: Low- and middle-income countries
MOU: Midwife obstetric unit
MRC: Maternity case record
NPO: Not-for-profit organisation
OTL: Outreach team leader
PHC: Primary healthcare
