# Supplementary material for: Facilitators and barriers to detection and treatment of depression, anxiety and experiences of domestic violence in pregnant women
Source: Sci Rep. 2023 Aug 1;13:12457. doi: 10.1038/s41598-023-36150-z (PMC10394005; doi:10.1038/s41598-023-36150-z)
Supplement: Supplementary file 1 — Supplementary Information 1. [file 41598_2023_36150_MOESM1_ESM.docx]

**Supplementary File 1**: Interview questions for healthcare workers

A. Understanding the role and attitudes of healthcare workers

1. Can you tell me about the work you do? (Probe: Where do you work – in the facility or in the community? What does your work entail? Who do you report to?)
2. Have you completed any training for working with people with mental health problems? What is this training/qualification called?
3. What experiences do you have in working with mental health problems?
4. Do you have any counselling experience? If so, describe this experience. What do you do when you counsel somebody?
5. What is the current care pathway for pregnant women with depression or anxiety?
6. Are you involved in the care of pregnant women with depression or anxiety? How are you involved?
7. What kind of support do you think depressed/anxious pregnant women and mothers need?
8. What do you think would be the best way to help pregnant women or mothers of young babies with depression or anxiety problems in your community?
9. When do they need help the most – during or after pregnancy, or both? If they need both, would the help they need be different at each time? If so, in what way?
10. Do you think that women commonly experience violence in your general community? Why do you think so? What kind of violence is most common?
11. Do you think pregnant women and mothers more likely (or less likely) to experience violence than other people? Why is this?
12. Do you think that many women who experience violence during their pregnancy or in the first year of motherhood attend this facility? Who do they disclose their experience of violence to?
13. How do your staff normally deal with this situation? Who would deal with it? What support or advice would they normally give to women in this situation?
14. Are you aware of any protocols or standard procedures that need to be followed to manage the situation of perinatal women who experience violence?
15. What is the current care pathway for women who experience domestic violence?
16. Are you involved in the care of pregnant women who experience domestic violence? How are you involved?
17. What kind of support do you think pregnant women and mothers who experience domestic violence need?
18. What do you think would be the best way to help pregnant women or mothers of young babies who experience domestic violence in your community?
19. When do they need help the most – during or after pregnancy, or both? If they need both, would the help they need be different at each time? If so, in what way?

B. Providing a routine screening service for pregnant women

1. Are pregnant women who attend this facility routinely screened for depression and anxiety? And for experiences of domestic violence?
2. What do you think about introducing routine screening for depression and anxiety, and for domestic violence at antenatal clinic visits? Do you think it is important to do so?
3. Who would be best positioned to do this screening as part of their routine antenatal check-up – would this be the midwives or nurses or other staff? What sort of support would they need to do this (eg training or supervision)?
4. If you were to design a service for women that could help with their depression/ anxiety and still formed part of their existing care, what would it look like? What health systems changes would be needed to make this work well?

C. Providing a counselling service for pregnant women

1. Do you know what mental health counsellors are? [If uncertain, explain]: They are people who work with people who are depressed or anxious to help them. They do this by listening to their problems and helping them to develop skills to deal with their problems and feel better.
2. What kind of counsellor is acceptable to pregnant women? (gender, age, qualification, culture, race, locality, relationship/ community relations, professional/lay person).
3. What do you think about counsellors doing home visits to do counselling for pregnant women? How might this be perceived by the women and communities?
4. How often should a counsellor visit the house, before and after the birth?
5. What skills would you like to learn if you were to counsel people who are depressed or anxious or experienced domestic violence?
6. What kind of support and supervision would you need in order to do counselling? How often would you like to meet with a supervisor/manager?
7. What obstacles do you see in delivering a counselling programme to patients at their homes?
8. What are the benefits of counselling women in their homes that you wouldn’t get if you counselled them at the clinic?
9. What obstacles do you see in delivering a counselling programme to patients at the clinic?
10. What are the benefits of counselling women at the clinic that you wouldn’t get if you counselled them at their homes?
