# Supplementary material for: Facilitators and barriers to detection and treatment of depression, anxiety and experiences of domestic violence in pregnant women
Source: Sci Rep. 2023 Aug 1;13:12457. doi: 10.1038/s41598-023-36150-z (PMC10394005; doi:10.1038/s41598-023-36150-z)
Supplement: Supplementary file 2 — Supplementary Information 2. [file 41598_2023_36150_MOESM2_ESM.docx]

**Supplementary File 2:** Interview questions for pregnant women

A. Understanding of depression and anxiety

Earlier you have mentioned a few things that relate to what we call ‘depression’ or ‘anxiety’, such as: (Refer to the EPDS and mention some factors they identified)

1. What other words would you use to describe the feelings I just mentioned? (Probe: thinking too much, deep sadness, feeling stressed, painful heart, worrying a lot, feeling down, nerves, etc.)
2. How would you know if a person has these problems (use their words). (Probe: How would they behave? What would they do or not do?)
3. How have these feelings changed your daily life? What problems has it caused in your life?
4. When did you first start to feel anxious/depressed (use their words)
5. What do you think caused you to feel this way? (Probe: illness, lack of money, death of someone close to you, lack of family support, lack of support from partner, unwanted pregnancy, stigma and discrimination, or witchcraft etc.)
6. Think about days when these feelings are really bad. Can you tell me what such a day is like?
7. Can you describe days when you feel better? (Probe: What makes it better? Was there anything you did yourself? Was there someone who helped you to feel better? If so, who, and what did they do?)
8. How does feeling like this (being depressed/anxious) change or affect the way you look after yourself? (Probe: In what ways do these feelings stop you from caring for yourself or stop you from looking for help for yourself?)
9. What do you think would make you feel more hopeful and confident?
10. Do you miss out on socialising and being part of groups because of these feelings? Can you give me some examples? Can you explain a bit more?
11. How does feeling depressed/anxious affect how you feel about having a baby? (Probe: What makes the situation difficult? Are there times when you didn’t or don’t feel so good about having a baby? How do you cope with these feelings?)
12. How do you think people with depression/anxiety are treated by health workers?
13. Have you ever had such an experience of being treated like this because of depression/anxiety?
14. What about your family and community, what do they think about people with depression/anxiety?

B. Understanding of domestic violence

Earlier I asked you whether you had experienced physical, sexual or verbal abuse during the last year, and you indicated that you had.

1. Can you tell me more about what happened? (Probe sensitively for circumstances, relationship with the perpetrator)
2. Do you feel safe in the place you are currently living or working in? (If the participant is not safe, ask if she would be willing to speak to a social worker who can assist with a protection order or other statutory procedures)
3. What has happened as a result of these events? (Probe for injuries, effects on mood, thoughts and behaviour and effects on personal relationships for her or other members of the family)
4. How are you planning to deal with this situation going forward? Do you need any assistance (for example I could refer you to a social worker or the police)?

C. Routine screening and counselling service for pregnant women

I’d like to now ask you about ways that we can help you or others like yourself.

1. Can you describe the help you got and how you felt about it? (Probe: Where did you go? Who helped you? What help were you given?)
2. Did this help change your feelings or how you managed the situation? Can you give me examples of how it made a difference?
3. Would you get help from this place/person/people again? Explain why?
4. How do you think we can find people with depression/anxiety/those who have experienced domestic violence, so that they can be helped? (probe: advertising, screening, more services)
5. Do you know about any other services offered by the clinic or hospital or any other organisation to help people with depression, anxiety or being a victim of domestic violence? What services are they?
6. What would make it easier or more difficult to access these services? And give examples to explain your answers.
7. Do you know what mental health counselling is? [If uncertain, explain: It is when a health worker tries to help people who are depressed or anxious. They do this by listening to their problems and helping them to develop skills to deal with their problems and feel better.] Have you ever had this type of counselling before?
8. Do you think that counselling could help you with your feelings? In what way could it help you?
9. If you could choose, who would you want to counsel you? A nurse, a community health worker, or an HIV counsellor?
10. If you could see a counsellor, would you rather see that person at the clinic or at your home? Explain why you gave that answer.
11. Do you think that counselling is best done alone or in a group with other people with similar feelings/ problems?
12. If someone came to your home. What would your family think? What would your community think?
13. Do you think it would be safe for that person to come and visit you?
14. If someone came to your home, do you have any private space where you can talk?
